# *OsPLS4* Is Involved in Cuticular Wax Biosynthesis and Affects Leaf Senescence in Rice

**DOI:** 10.3389/fpls.2020.00782

**Published:** 2020-06-11

**Authors:** Dahu Zhou, Ting Li, Yaolong Yang, Ziyang Qu, Linjuan Ouyang, Zhishu Jiang, Xiaoli Lin, Changlan Zhu, Liyuan Peng, Junru Fu, Xiaosong Peng, Jianmin Bian, Wenbang Tang, Jie Xu, Haohua He

**Affiliations:** ^1^Southern Regional Collaborative Innovation Center for Grain and Oil Crops, Hunan Agricultural University, Changsha, China; ^2^Key Laboratory of Crop Physiology, Ecology and Genetic Breeding, Ministry of Education, College of Agronomy, Jiangxi Agricultural University, Nanchang, China; ^3^State Key Laboratory of Rice Biology, China National Rice Research Institute, Hangzhou, China

**Keywords:** chloroplast, leaf senescence, cuticular wax, fatty acid synthesis, chilling stress, *Oryza sativa*

## Abstract

Leaf senescence is one of the most common factors that affects the growth and yield of rice. Although numerous genes affecting leaf senescence have been identified, few involved in cuticular wax synthesis have been described for rice premature leaf senescence. Here, we cloned and characterized *Premature Leaf Senescence 4 (PLS4)* in rice (*Oryza sativa*), which encodes a putative 3-oxoacyl-reductase in the fatty acid biosynthetic pathway. Subcellular localization of OsPLS4 was observed in the chloroplast. A single nucleotide substitution in *OsPLS4* reduced leaf cuticular wax, and the expression levels of most wax biosynthesis-associated genes were downregulated. TEM showed chloroplast development were defective in the *pls4* mutant. Further investigation revealed that the chlorophyll (Chl) content was reduced in the *pls4* mutant compared with the WT and that the photosynthesis rate was lower, which caused ROS dramatic accumulation at the heading stage. These results confirmed premature leaf senescence in *pls4* plants. Cold treatment indicated that the mutant was more sensitive than the WT was to cold stress. Together, all the above results indicate that the *OsPLS4* mutation affects cuticular wax biosynthesis and chloroplast development in rice, causing reduced cuticular wax and premature leaf senescence.

## Introduction

Rice (*Oryza sativa*) is one of the most important cereal crop species worldwide and is consumed by more than half of the global population. Leaf senescence is the final process of leaf development, during which time intracellular organelles and macromolecules are actively destabilized to relocate nutrients into developing tissues or storage organs (Sakuraba et al., 2016). Premature leaf senescence affects rice yields and quality by reducing photosynthetic efficiency and the accumulation of nutrients (Yang et al., 2016; Mao et al., 2017). Hence, understanding the process of premature leaf senescence and the relevant molecular mechanisms could be beneficial for crop breeding.

A large number of senescence-associated genes (SAGs) have been identified, and these genes seem to function in various biological processes, such as the degradation of chloroplasts, the accumulation of reactive oxygen species (ROS), and biotic and abiotic stress responses (Jiao et al., 2012; Liang et al., 2014; Sakuraba et al., 2015; Yang et al., 2016; Leng et al., 2017a). Chlorophyll (Chl) is an essential molecule for the harvest of light energy during photosynthesis. A large number of genes involved in either Chl biosynthesis or degradation have been verified to respond to leaf senescence (Horie et al., 2009; Sato et al., 2009; Yamatani et al., 2013; Yasuhito et al., 2013). Moreover, Chl degradation accelerates leaf yellowing, which is the most notable sign of leaf senescence. Several genes are associated with Chl degradation *OsNOL* and *OsNYC1* (Kusaba et al., 2007; Sato et al., 2009), which encode two short-chain dehydrogenases/reductases that serve as Chl b reductases; *OsNYC3* (Morita et al., 2010), which encodes an α/β hydrolase-fold family protein; *OsNYC4* (Yamatani et al., 2013), whose product is involved in thylakoid formation and chloroplast precursors; *OsPAO*, which encodes a pheophorbide oxygenase; and *OsRCCR1* (Tang et al., 2011), which encodes a reductase of Chl-like catabolites. Several leaf color-associated mutations have been reported in rice, such as, GATA transcription factor 1 has been shown to regulate chloroplast biogenesis positively (Hudson et al., 2013). The *pale green leaf* encodes an oxygenase required for the biosynthesis of Chl, which has also been shown to play key roles in rice yields and quality (Yang et al., 2016). Additionally, premature leaf senescence is related with excess ROS overproduction. If ROS accumulation is not reduced within interior plant cells in a timely and effective manner, the cell membranes can disrupted, protein and chloroplast degradation can be promoted, ultimately resulting in early leaf senescence. For example, the *LTS1* gene encodes a nicotinate phosphoribosyltransferase (NaPRT1), which is involved in NAD biosynthesis, and a point mutation in *OsNaPRT1* leads to hydrogen peroxide (H_2_O_2_) accumulation in leaves and triggers premature leaf senescence (Wu et al., 2016). The *pls3* mutant presents a reduced Chl content and an increased H_2_O_2_ content, the *OsMTS1* encodes a methyltransferase, is required for melatonin biosynthesis in rice, and disruption of *OsMTS1* can trigger premature leaf senescence (Hong et al., 2018).

Plant cuticular wax serves as an essential barrier for preventing abiotic and biotic stress damage, such as exposure to dust, pathogens and high/low temperature (Aharoni et al., 2004; Monica et al., 2005; Weidenbach et al., 2014). Cuticular wax is mainly composed of mainly very-long-chain fatty acids (VLCFAs; chain length ranging from C20 to C34 carbons) and their derivatives (including aldehydes, alcohols, alkanes, ketones, and esters) (Samuels et al., 2008; Haslam and Kunst, 2013). Cuticular wax biosynthesis is very complex and begins with *de novo*-synthesized C16 and C18 fatty acids within the plastids. C16 and C18 fatty acids are then elongated to VLCFAs by the fatty acid elongase (FAE) complex, which comprises β-ketoacyl-CoA synthase (KCS), β-ketoacyl-CoA reductase (KCR), β-hydroxy acyl-CoA dehydratase, and enoyl-CoA reductase, in the endoplasmic reticulum (ER). VLCFA elongation involves a four-step reaction cycle. First, the condensation of C16 and C18 acyl-CoA is catalyzed by KCS, yielding β-ketoacyl-CoA; second, the reduction of β-ketoacyl-CoA is catalyzed by KCR; third, the resulting β-hydroxy acyl-CoA is dehydrated by β-hydroxy acyl-CoA dehydratase (HCD); and fourth, the enoyl acyl-CoA is reduced by enoyl-CoA reductase (ECR) (Benning, 2009; Lippold et al., 2012; Li et al., 2016). 3-oxoacyl-(acyl carrier protein[ACP]) reductase (OAR), which catalyzes the first reduction step in each cycle of fatty acid elongation in plants (Slabas et al., 1992; Winter et al., 1997; O’Hara et al., 2007), belongs to a very large family of enzymes, the short-chain alcohol dehydrogenase/reductase (SDR) family, whose members carry out a series of reduction and dehydrogenation reactions via NADH or NADPH (Persson et al., 2003; Wickramasinghe et al., 2006; Kavanagh et al., 2008). The functions of SDR encompass many aspects of primary (lipid synthesis and Chl biosynthesis or degradation) and secondary (terpenoids, steroids, phenolics, and alkaloids) metabolism (Moummou et al., 2012). Several wax biosynthesis genes have previously been identified in rice by the characterization of *wax crystal-sparse leaf* (*wsl*) mutants. *OsWSL1* encodes a KCS that is involved in the biosynthesis of cuticular waxes on rice leaves (Yu et al., 2008). *OsWSL2* encodes a homolog of the *Arabidopsis thaliana* genes (*CER3/WAX2/YRE/FLP1)* and the maize gene *GL1*, which is associated with the elongation of VLCFAs (Mao et al., 2012). *OsWSL3* encodes a KCR that affects cuticular wax biosynthesis in rice (Gan et al., 2016). *OsWSL4* is predicted to encode a KCS that is homologous to the product of *Arabidopsis CER6* (Wang et al., 2017). *OsWSL5* is predicted to encode a cytochrome P450 family member CYP96B5, which is involved in the formation of epidermal wax crystals on rice leaf affecting drought sensitivity (Zhang et al., 2020). *OsWS1* is involved in wax biosynthesis and is regulated by *osa-miR1848* (Xia et al., 2015). Although several wax biosynthesis-related genes have been cloned in rice, few wax related mutants have been reported to be associated with early leaf senescence. In particular, some enzymes related to the wax synthesis pathway regulate plant senescence, which has rarely been studied in rice. However, in *Arabidopsis thaliana*, two genes (*PES1 and PES2*) belong to the esterase/lipase/thioesterase family of acyltransferases, which are involved in fatty acid phytyl ester synthesis in chloroplasts, a process involved in maintaining the integrity of the photosynthetic membrane during abiotic stress and senescence (Felix et al., 2012). This study indicated these genes may maintain the integrity of the biological membrane to defense stress and senescence in plants.

In this study, we isolated and characterized a rice *premature leaf senescence 4* (*pls4*) mutant. Using map-based cloning strategy, we found that a single nucleotide substitution in *OsPLS4* was responsible for the phenotypic variation, and the *OsPLS4* mutation resulted in cuticular wax decrease. *OsPLS4* is predicted to encode a 3-oxoacyl-ACP reductase, which participates in the reduction reaction of the first step of fatty acid biosynthesis in chloroplasts. We performed detailed functional analyses to explore the role of *OsPLS4* in leaf senescence in rice on the basis genetic, physiological and biochemical approaches. Our results demonstrated that *OsPLS4* plays an essential role in cuticular wax synthesis chilling stress in rice.

## Materials and Methods

### Plant Materials and Growth Conditions

We obtained a premature leaf senescence (*pls4*) mutant from a mutant population generated by EMS treatment of ZH11 (*O. sativa japonica*). An F_2_ mapping population was generated from a cross between the *pls4* mutant and rice variety TN1(*O. sativa indica*). All plants were grown in a paddy field at Jiangxi Agricultural University, Nanchang, Jiangxi Province, China, and at Sanya, Hainan Province, China.

### Fine Mapping and Isolation of *PLS4*

We crossed *pls4* with TN1 and used the F_2_ population for gene mapping. For primary mapping, BSA was performed with DNA pools from 30 F_2_ individuals with the *pls4* phenotype and from 30 F_2_ individuals with the wild type phenotype. The initial localization was determined via 182 SSR markers from the 12 chromosomes of rice^1^. Afterward, 985 recessive individuals with the premature leaf senescence(the mutant phenotype) were selected from the F_2_ population to fine map the *PLS4* locus. In the *PLS4* primary mapped region, we developed InDel markers on the basis of sequence comparisons between the genomic sequences of *Nipponbare* and 9311 to narrow down. All primer sequences used for gene mapping are shown in Supplementary Table S2. The products were subsequently separated on 8% polyacrylamide gels.

### Functional Complementation of the *pls4* Mutant

For the complementation of the *pls4* mutant, a 5221-bp genomic DNA fragment that included the *PLS4* coding region along with the upstream and downstream sequence were introduced into a pCAMBIA1300 binary vector to generate the transformation construct, yielding pCAMBIA1300-*OsPLS4*. pCAMBIA1300-*OsPLS4* was transformed into the calli generated from the seed embryos of the *pls4* mutant by *Agrobacterium*-mediated transformation. The primers used are listed in Supplementary Table S1.

### Scanning and Transmission Electron Microscopy

Tissue sections from the *pls4* mutant and the same region in WT were selected at the heading stage for SEM and TEM analysis.

SEM was performed as previously described. The detached leaves were pre-fixed in fixation buffer (2.5% glutaraldehyde-sodium in 100 mM phosphate buffer) at room temperature and post-fixed in 1% OsO_4_ at 4°C. The samples were dehydrated in a series of ethanol and dried with a critical point dryer. Subsequently, the prepared samples were coated with platinum by a sputtering instrument and examined by SEM (S-4800, Hitachi, Japan) at an accelerating voltage of 10 kV.

For TEM analysis, the pre-fixed tissue segments were processed as previously described, embedded in paraffin wax, mounted, and observed under an H-7650 transmission electron microscope (Hitachi, Japan) (Broun et al., 2004).

### Quantitative Real-Time PCR and Promoter-GUS Analysis

Total RNA was extracted from rice tissues using a Takara Plant MiniBEST RNA Extraction Kit (Takara, Japan), and cDNA was synthesized by PrimeScript^TM^ II reverse transcriptase (Takara, Japan) according to the manufacturer’s instructions. qRT-PCR was performed using a 2 × SYBR Green PCR Master Mix (Applied Biosystems, United States) on a 7500 Real-Time PCR System (Applied Biosystems, United States) in a reaction volume of 20 μL. The *OsUBQ (Ubiquitin)* gene was used as a control.

For the promoter-GUS assay, the 2040-bp genomic DNA fragment was amplified from *PLS4* promoter region upstream of WT genomic DNA via primers (Supplementary Table S2), the resulting fragment was introduced into a pCAMBIA1305-GUS vector upstream of the GUS reporter gene. Rice transformation and histochemical analysis were performed as previously described (Tian et al., 2013).

### Subcellular Localization and Promoter Fusions

To produce the subcellular localization of *PLS4*, the *PLS4* coding DNA sequence without a termination codon was amplified and cloned into a subcellular localization pCaMV35S-GFP vector to fuse *PLS4* to an enhanced green fluorescent protein (eGFP) (Bottanelli et al., 2012). The control construct (p35S:GFP) and the fusion constructs (p35S:PLS4-GFP) were subsequently transiently expressed into tobacco (*Nicotiana benthamiana*) epidermal leaf cells as described previously (Batoko et al., 2000). The cells were then examined under a confocal fluorescence microscope (Leica TCS SP5, Germany) after 48 h of incubation. The GFP and Chl fluorescence at 522 and 680 nm was recorded respectively.

To assess the subcellular localization of *PLS4*, its coding sequences were subcloned into a *Spe*I- and *Bam*HI-digested pAN580 vector to produce a C-terminal GFP fusion construct driven by the constitutive 35S promoter. The constructs were then cotransformed together with a chloroplast marker fusion construct into rice protoplasts as described previously (Aggarwal et al., 2014). The transformed cells were detected by confocal microscopy (Olympus FV1000 MPE) and images were captured at 488 nm for GFP excitation and at 534 nm for red fluorescent protein (RFP) excitation. The primers used are listed in Supplementary Table S2.

### Leaf Cuticular Wax Components Analysis

Cuticular waxes were extracted from mature expanded and the same region blade leaves at the heading stage and measured as described previously (Mao et al., 2012). Briefly, by immersing leaves from the flag at the heading stage into 30 mL of n-hexane at 67°C for 30 s, with 50 μg of *n*-tetracosane used as an internal standard. The *n*-hexane was then evaporated under gaseous N_2_ and the residue was derivatized with 100 μL of bis-*N,N*-(trimethylsilyl) trifluoroacetamide (BSTFA, Sigma, St. Louis, MO, United States) and 100 μL of pyridine for 60 min at 70°C. All wax samples were analyzed with an Agilent (Santa Clara, CA, United States) 7000C gas chromatography-mass spectrometry (GC-MS) device on a 30 m HP-1MS column. The column was operated with helium as the carrier gas and with splitless injection at 250°C. The oven temperature was increased from 50 to 200°C at 20°C min^–1^, maintained for 2 min at 200°C, increased at 2°C min^–1^ to 320°C, and then maintained at 320°C for 14 min. The total amount of cuticular wax was expressed per unit area of the leaf surface. The leaf area was measured with an LI-3000C Portable Area Meter (LI-COR Biosciences, Lincoln, NE, United States). All experiments consisted of five biological replicates.

### Measurement of the Chl Content and Photosynthetic Rate

Chl was extracted from the fully expanded leaves of rice plants at four development stages and at the heading stage in two independent complementation lines measured according to previous methods (Porra et al., 1994). Briefly, 0.1 g of leaf tissue was immersed in 25 mL of 95% alcohol for 24 h in darkness. Afterward, the Chl content was measured with a DU800 visible spectrophotometer (Beckman, CA, United States) at 649 and 665 nm.

The photosynthetic rates were measured at the heading stage of WT and *pls4* mutant from 10:00–12:00 h on a sunny day by the portable photosynthetic measurement device LI-6400 (LI-COR Biosciences, Lincoln, NE, United States). All experiments consisted of three biological replicates.

### Rate of Water Loss and Chl Efflux

The leaf water-loss rate was measured for detached leaves of plants at the heading stage of WT and *pls4* mutant. The same parts of the flag leaves were cut into 5cm lengths pieces and placed in a Petri dish. In the dark, the leaves were weighted every 1 h. With respect to Chl leakage, 0.1 g of leaf blades tissue of plants at the heading stage was immersed in 25 mL of 95% alcohol. A series of 100 μL aliquots were collected at 1, 2, 3, 4, 5, 6, 7, 8, 9, and 10 h and subjected to spectrophotometry (absorption measured at 647 and 664 nm) to quantify the amount of Chl that was leached. All experiments consisted of three biological replicates.

### Histochemical Analysis, Determination of H_2_O_2_ and MDA Contents, and Detection of CAT Activity

The accumulation of the O_2_^–^ was measured via NBT (0.5 mg mL^–1^ in 10 mM potassium phosphate buffer, pH 7.6). The staining and bleaching of the leaves were performed as previously described (Yang et al., 2016). The H_2_O_2_ and MDA contents and CAT activity were determined by the use of Suzhou Comin Biotechnology kits via spectrophotometry with respect to the H_2_O_2_ content, H_2_O_2_ and titanium sulfate form a yellow titanium peroxide complex with characteristic absorption at 415 nm (Code: H_2_O_2_-1-Y, Comin); with respect to the MDA contents, the MDA is combined with thiobarbituric acid (TBA) to form a red product, which has maximum absorption peak at 532 nm (Code: MDA-2-Y, Comin); and the CAT activity was measured normally (Code: CAT-1-Y, Comin). All experiments consisted of three biological replicates.

### Chilling Treatment

To test chilling tolerance, the seedlings of WT and *pls4* were treated at 4°C for 72 h. Afterward, they were moved to a temperature-controlled greenhouse that had a 30°C/25°C day/night cycle for recovery for 7 days. After treatment at 4°C, leaves of the seeding were removed every 3 h to measure the expression of genes related to cold tolerance.

### Investigation of Agronomic Traits

The plant height and tiller number of different rice lines were measured under field conditions. When the rice plants grew to maturity in the field, their height, tiller number, panicle length, number of grains per panicle, seed setting rate and 1000-grain weight were measured via conventional methods, with five biological replicates per WT and mutant plants.

## Results

### Characterization of the *pls4* Mutant

**FIGURE 1:**
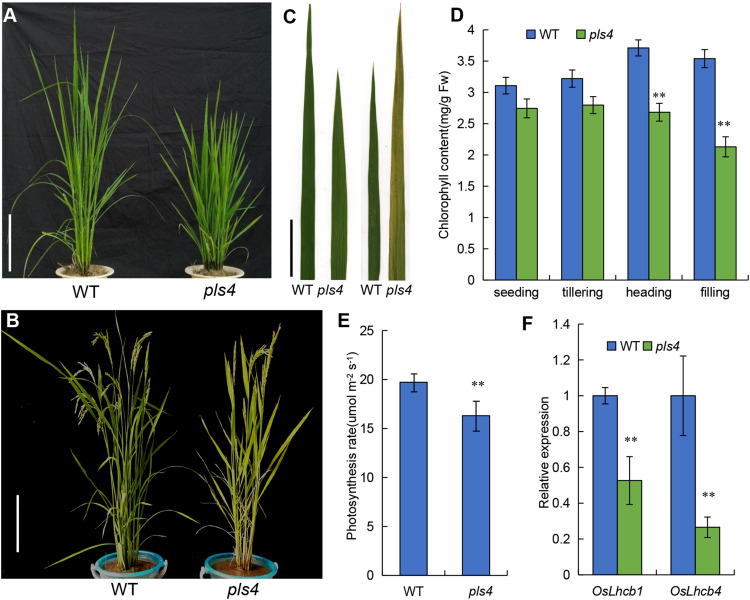
Characteristics of the WT and *pls4* mutant. **(A,B)** Phenotypes of the WT and *pls4* mutant at the tillering and heading stages. Bars = 20 cm. **(C)** The leaves of the WT and *pls4* mutant at the tillering and heading stages. Bars = 5 cm. **(D)** Chl content in the leaves of *pls4* and the WT at four growth stages. Fw, fresh weight. **(E)** Determination of the photosynthesis rate of the WT and *pls4* mutant at the heading stage. **(F)** Expression levels of *OsLhcb1* and *OsLhcb4*; *OsLhcb1*, light-harvesting Chl a/b-binding protein of photosystem II (PSII) (*LOC_Os10g41780*); *OsLhcb4 (LOC_Os07g37240).* The data presented are the means ± SDs of three biological replicates. **P < 0.01 (Student’s *t*-test).

We isolated a premature leaf senescence mutant described as *pls4* from a mutant library of the japonica rice variety Zhong Hua 11 (ZH11), which was generated by ethyl methyl sulfonate (EMS) treatment. The *pls4* mutant exhibited multiple different phenotypes from wild-type (WT) ZH11. At the tillering stage, compared with those of the WT, the plant height and size of the *pls4* mutant were reduced, but there was no obvious difference in leaf color (Figure 1A); compared with that in the WT, the Chl content in the mutant moderately decreased, but the difference was not significant (*P* > 0.05) (Figure 1D). However, after heading, the leaves color changed from green to yellow and some brown spots were visible (Figures 1B,C), and the Chl content rapidly decreased in *pls4* (Figure 1D). Which are typical phenomena associated with leaf senescence.

We found that the photosynthetic rate was significantly lower in the flowering leaf of the *pls4* mutant than in the WT (Figure 1E). *OsLhcb1* and *OsLhcb4* are essential to the photosynthetic system of rice, and their expression was also detected. The qRT-PCR results showed that the expression of both *OsLhcb1* and *OsLhcb4* was strongly suppressed in the *pls4* mutant compared with the WT; the *OsLhcb1* and *OsLhcb4* expression in the *pls4* mutants was only approximately one-half and one-quarter of that in the WT plants, respectively (Figure 1F).

Because photosynthesis and leaf senescence in the late stage strongly impact on yield formation, several agronomic traits, including plant height, tiller number, panicle length, grain number per panicle, seed setting rate and 1000-grain weight, were investigated. The results showed that plant height, tiller number, panicle length and grain number per panicle were moderately affected in the *pls4* plants, while the seed setting rate and 1000-grain weight significantly decreased. Both the seed setting rate and 1000-grain weight in the mutant were only approximately 70% of those in the WT (Supplementary Figure S2 and Supplementary Table S3).

### Map-Based Cloning of *OsPLS4*

To identify whether the premature-senescence phenotype of *pls4* is caused by a single gene or multiple genes, reciprocal crosses of *pls4* with common cultivars (including Nip, TN1 and 93-11) were carried out. The obtained F_1_ plants from all the crosses displayed a normal phenotype, and none of them showed a Chl degradation-type phenotype (Supplementary Figure S1). Segregation of the traits was observed in all F_2_ populations, and the ratio of WT plants to plants with a premature leaf senescence phenotype was close to 3:1 according to a χ^2^ test (Supplementary Table S1). These results indicated that the premature leaf senescence phenotype of *pls4* is a qualitative trait that is controlled by a single recessive gene.

**FIGURE 2:**
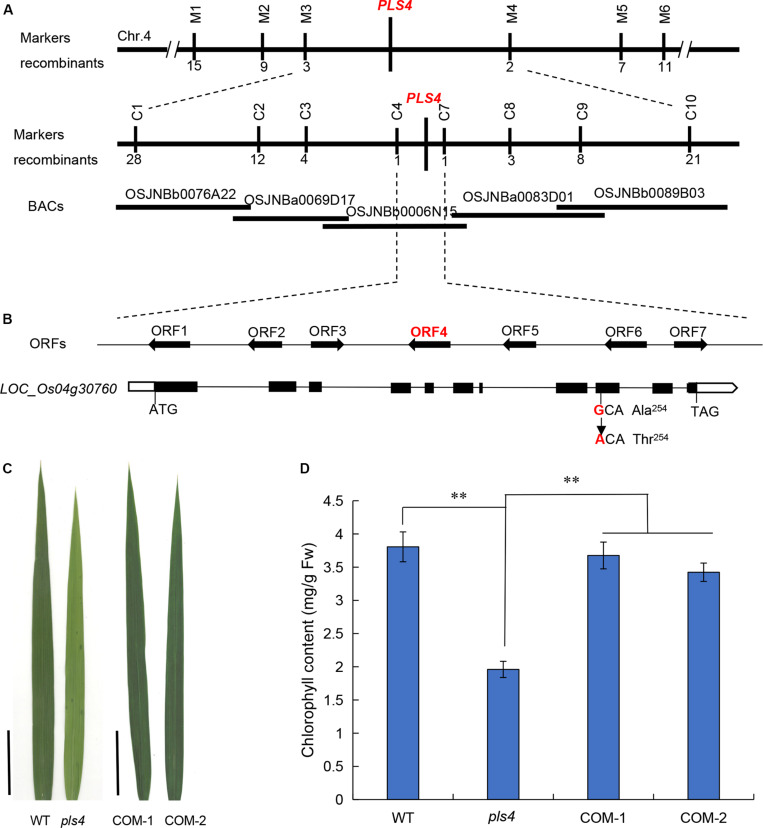
Map-based cloning of the *OsPLS4* gene. **(A)** Fine mapping of *PLS4* further localized the mutation to a 52-kb genomic region between C4 and C7 on chromosome 4. The number of recombination events between marker and *pls4* phenotype. The target region contains 7 candidate ORFs. **(B)** Schematic of *PLS4.* The black rectangles represent exons, and the position of the SNP is indicated in the ninth exon of *LOC_Os04g30760*; the SNP resulted in the mutation of the encoded protein at position 254 from alanine (Ala) to threonine (Thr). **(C)** The phenotype of the flag leaves in the transplanted lines, COM-1,-2: two independent complementation plants; Bars = 5 cm. **(D)** The flag leaf Chl content of the WT, *pls4* and two COM independent lines at the heading stage. The data presented are the means ± SDs of three biological replicates. **P < 0.01 (Student’s *t*-test).

To isolate *OsPLS4*, the F_2_ population from the cross between the *pls4* mutant and TN1 were selected for gene mapping. Bulked segregant analysis (BSA) was used for linkage analysis and primary mapping with 182 simple sequence repeat (SSR) markers. The results revealed that several SSR markers on chromosome 4 were co-segregated with the *pls4* phenotype, and the locus was mapped between markers M3 and M4 (Figure 2A). Fine mapping was subsequently performed with InDel markers according to the difference in the primary markers between *Nipponbare* and 93-11, and the location of the *OsPLS4* locus was narrowed to a 52-kb interval between C4 and C7, which included 7 predicted open reading frames (ORFs) (Figure 2A). DNA sequencing analysis of the whole region revealed that a single nucleotide substitution (G-A) was present within the ninth exon of the fourth ORF, *LOC_Os04g30760* (Figure 2B). For the remainder of this paper, *pls4* will continue to be used as the mutant line name, and *OsPLS4* will be used as the gene name. The mutation of *LOC_Os04g30760* within *pls4* leads to an amino acid change from alanine (Ala) to threonine (Thr) at the 254th amino acid residue (Figure 2B). These results suggested that the mutation of *LOC_Os04g30760* was possibly responsible for the abnormal phenotype of *pls4*.

To investigate the linkage between the *pls4* phenotype and the mutation of *LOC_Os04g30760*, we transformed the mutant with the pCAMBIA1300 binary vector (COM), which contained a genomic fragment encompassing the WT gene, including its native promoter and a 957-bp downstream sequence. Twenty-three independent T_0_ transformants were obtained, and the leaf premature-senescence phenotype was completely restored in the transgenic plants (Figure 2C and Supplementary Figure S3). Moreover, the COM plants were nearly the same as the WT plants in terms of their Chl content and *OsPLS4* expression level (Figure 2D and Supplementary Figure S4). In summary, these results indicated that the cloned candidate gene *LOC_Os04g30760* was indeed responsible for the *pls4* phenotype.

The ORF of *OsPLS4* is predicted to encode a protein of 319 amino acids composing 3-oxoacyl-reductase. Compared with homologous proteins from other species (sorghum [*Sorghum bicolor*], maize [*Zea mays*], Brachypodium [*Brachypodium Beauv*], and A*rabidopsis*), nearly 86.69% of amino acids throughout the protein coned by *OsPLS4* are conserved, as revealed by ClustalW alignment (Supplementary Figure S5). Additionally, the mutated amino acid residue within *OsPLS4* is truly conserved in monocotyledons (i.e., sorghum, maize and Brachypodium) (Supplementary Figure S5). Molecular phylogenetic analysis was performed with homologous protein sequences of *OsPLS4* from various plant species, which revealed that monocotyledons are closely related (Supplementary Figure S6). The results of the BLAST sequence and phylogenetic analyses demonstrate that *OsPLS4* is important and conservative in monocotyledonous plants and that the Ala mutation in this study is very important for *OsPLS4* function in rice.

### Expression Pattern and Subcellular Localization of *OsPLS4*

**FIGURE 3:**
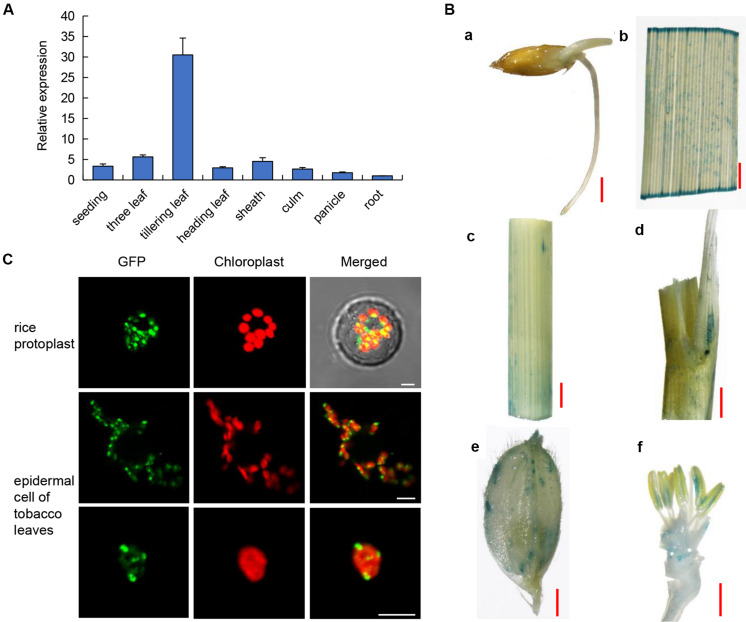
Expression patterns and subcellular localization. **(A)** Expression pattern of *OsPLS4.* Expression in various organs, including the leaves, roots, culms, leaf sheaths, and panicles at the heading stage in the WT. The data presented are the means ± SDs of three biological replicates. **(B)** Spatial expression pattern of the *OsPLS4* gene in transgenic rice plants harboring the *OsPLS4* promoter fused to the GUS gene. **(a)** germinating seed; **(b)** spikelet; **(c)** stem; **(d)** leaf sheath; **(e)** tillering leaf; **(f)** anther. Bars = 1 mm. **(C)** Subcellular localization of *OsPLS4*-GFP fusion constructs. The transient expression of *OsPLS4-GFP* fusion constructs in rice protoplasts and epidermal cells of *N. benthamiana* leaves was imaged by a confocal microscope. Bars = 5 nm.

To detect the expression pattern of *OsPLS4*, we used qRT-PCR to analyze its transcriptional level in several tissues, including the roots, culms, leaves, sheaths and panicles, at several stages. The results indicated that *OsPLS4* was widely expressed in different rice tissues. *OsPLS4* was expressed at the lowest level in the roots and was expressed at the highest level in green tissues. Moreover, even in the leaves, the transcript level of *OsPLS4* was different at the different developmental stages. *OsPLS4* had the highest expression level at the tillering stage approximately 30 times that in the roots and more than 6 times that in the leaves at other stages (Figure 3A). Analysis of *OsPLS4* expression in the tillering leaves of WT and *pls4* mutant, the result showed no significant difference (Supplementary Figure S4). The *OsPLS4* gene expression pattern was further confirmed by β-glucuronidase (GUS) assays. GUS expression was detected in the roots, leaf blades, sheaths, stems, inflorescences, glumes, and anthers, but not in the stigma papillae, which is consistent with the results of the RT-PCR and qPCR analyses (Figure 3B). Strong GUS signals were detected in the tillering leaves (Figure 3Bb). *OsPLS4* was highly expressed in green tissues, indicating that *OsPLS4* plays an essential roles at the tillering stage in rice.

The subcellular localization of OsPLS4 was also explored to further study its functions. We fused GFP to the C-terminus of OsPLS4 and then transiently expressed the construct into both rice protoplasts and tobacco leaf epidermal cells. The same result was obtained in both rice and tobacco, and the GFP signal of the fused protein overlapped with that of chloroplast auto-fluorescence. We found that OsPLS4 was located specifically in the chloroplast (Figure 3C). This result indicated that OsPLS4 is involved in fatty acid biosynthesis in the chloroplast.

### *OsPLS4* Regulates Leaf Cuticular Wax Synthesis

**FIGURE 4:**
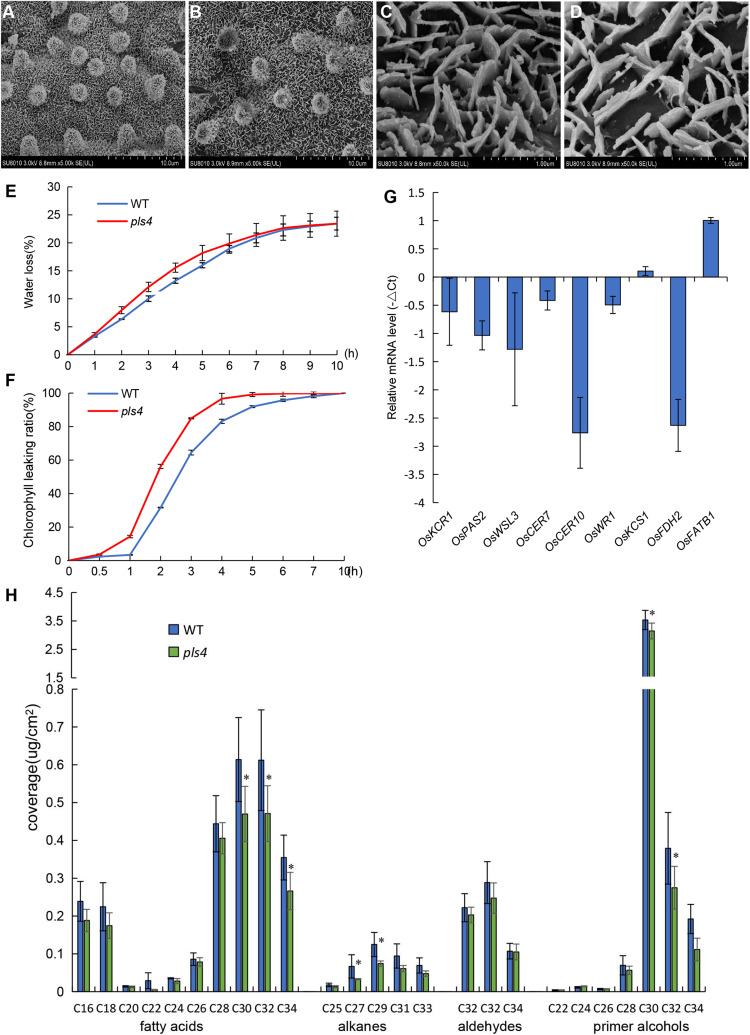
Analysis of cuticular waxes in the leaves of the WT and *pls4* mutant. **(A–D)** Cuticular wax crystals from the leaves of the WT **(A,C)** and *pls4* mutant **(B,D)** at the heading stage by SEM. Bars = 50 μm **(A,B)**, 5 μm **(C,D)**. **(E)** Rate of water loss from detached leaves of the WT and *pls4* mutant. **(F)** Chl leakage assays of leaves of the WT and *pls4* mutant. **(G)** Relative expression of a set of genes associated with wax synthesis in the leaves of WT and *pls4* mutant at the tillering stage. The data presented are the means ± SDs of three biological replicates. **(H)** Cuticular wax composition on the leaf surfaces of the WT and *pls4* mutant analyzed by GC-MS. Asterisks denote significant differences from the WT. The data presented are the mean ± SD from five biological replicates. *P < 0.05 (Student’s *t*-test).

*OsPLS4* encodes a putative 3-oxoacyl-reductase, which is a key enzyme involved in fatty acid biosynthesis found in many plant and bacterial species. In plants, fatty acids are a major component of cuticular wax. Therefore, leaf cuticular wax in both WT and *pls4* was evaluated. Scanning electron microscopy (SEM) assay revealed that the platelet-like wax crystals were distributed at a high density on the surface of WT leaves (Figures 4A,C), whereas wax crystals were significantly reduced on the surface of the mutant leaves and were sparsely distributed (Figures 4B,D). Moreover, the contents of all wax components were measured with gas chromatography-mass spectrometry (GC-MS). Compared with those of WT leaves, the total wax loads of *pls4* leaves were significantly reduced by 19.3% (*P* < 0.05, Table 1). Further analysis revealed that the contents of the four major components (fatty acids, aldehydes, primer alcohols, and alkanes) were reduced in the mutant; fatty acids, primer alcohols and alkanes were significantly reduced (*P* < 0.05) by 24.4, 16.4, and 37.8%, respectively (Figure 4H and Table 1).

**TABLE 1:** Leaf blade of the heading stage cuticular wax composition and loads in WT and *pls4* mutant.

Compound class	WT (μg/cm^2^)	*pls4* (μg/cm^2^)
Fatty acids	2.79 ± 0.39	2.11 ± 0.20*
Aldehydes	0.62 ± 0.09	0.56 ± 0.07
Primer alcohols	4.20 ± 0.45	3.51 ± 0.23*
Alkanes	0.37 ± 0.12	0.23 ± 0.02*
Total wax	8.09 ± 1.15	6.53 ± 0.48*

Data are mean ± SE from five biological replicates. *P < 0.05 (Student’s *t*-test).

Leaf permeability is strongly influenced by the quantity of cuticular wax. Thus, the rate of water loss and Chl leakage were measured in *pls4*. The water loss rate from detached leaves was faster in the *pls4* mutant than in the WT (Figure 4E), and the Chl leakage assay showed that Chl was more readily detected from *pls4* leaves than from WT leaves (Figure 4F). These results indicate that the cuticle of *pls4* is more permeable than that of the WT due to a scarcity of wax.

To investigate the molecular basis for the sparse crystals on the leaves, we measured the expression levels of wax synthesis-related genes (i.e., *OsKCR1, OsPAS2, OsWSL3, OsCER7, OsCER10, OsWR1, OsKCS1, OsFDH2*, and *OsFATB1*). Compared with that in the WT, with the exception of the expression of *OsKCS1* and *OsFATB1*, the expression of these genes in *pls4* was downregulated (Figure 4G). The expression of *OsCER10* and *OsFDH2* was strongly suppressed in *pls4*, and their expression levels were up to 6 times lower than those in the WT (Figure 4G). *OsKCS1* and *OsFATB1* encode a β-ketoacyl-CoA synthase and a fatty acyl-ACP thioesterase B, which are both involved in VLCFAs synthesis. As to why the expression of *OsKCS1* and *OsFATB1* genes upregulated, the regulatory mechanism is still unclear. These results demonstrated that *OsPLS4* functions in cuticular wax biosynthesis, not only as an enzyme but also as a regulator of other enzymes involved this process.

### Leaf Senescence Is Exacerbated in the *pls4* Mutant

**FIGURE 5:**
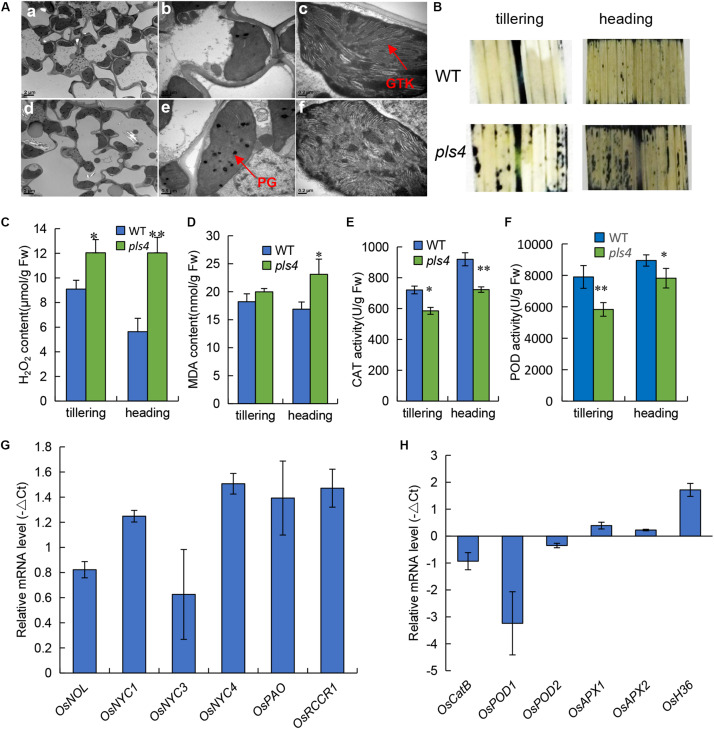
Leaves of *pls4* exhibit a severe senescence phenotype under natural conditions. **(A)** TEM analysis of the flag leaves of the WT and *pls4* mutant at the heading stage. GTK, granum thylakoid; PG, plastoglobule. Bars = 2 μm **(a,d)**, 0.5 μm **(b,e)**, 0.2 μm **(c,f)**. **(B)** Accumulation of O_2_^–^ radicals in naturally senescence leaves, visualized by staining with NBT. **(C–F)** H_2_O_2_ and MDA contents, CAT and POD activity in the WT and *pls4* mutant at the tillering and heading stages. The data presented are the means ± SDs of three biological replicates. *P < 0.05; **P < 0.01 (Student’s *t*-test). FW, fresh weight. **(G)** Changes in transcript levels of Chl degradation-associated genes in the leaves of the WT and *pls4* mutant at the tillering stage. The genes are as follows: *OsNOL* (*LOC_Os03g45194*) and *OsNYC1* (*LOC_Os01g12710*), two short-chain dehydrogenase/reductases, represent Chl b reductases; *OsNYC3*, which encodes an α/β hydrolase-fold family protein (*LOC_Os06g24730*); *OsNYC4*, which encodes thylakoid formation 1, and is involved with chloroplast precursors (*LOC_Os07g37250*); *OsPAO*, which encodes a pheophorbide oxygenase (*LOC_Os03g05310*); and *OsRCCR1*, which encodes reductase of Chl-like catabolites (*LOC_Os10g25030*). **(H)** Relative expression of a set of genes associated with senescence in the WT and the *pls4* mutant. The genes include the following: *OsCatB*, catalase (*LOC_Os06g51150*); *OsPOD1* (*LOC_Os01g22370*) and *OsPOD2* (*LOC_Os03g22010*), two peroxidases; *OsAPX1* (*LOC_Os03g17690*) and *OsAPX2* (*LOC_Os07g49400*), two ascorbate peroxidases; and *OsH36*, aminotransferase, senescence-induced protein (*LOC_Os05g39770*). The data presented are the means ± SDs of three biological replicates.

The *pls4* plants exhibited obvious early leaf senescence at the heading stage (Figures 1B,C). Compared with those in the WT plants, TEM revealed that the number and size of chloroplasts were dramatically reduced in the flag leaves of the *pls4* during the heading stage (Figures 5Aa,d). Moreover, in the *pls4* mutant, the granum thylakoids (GTKs) were arranged disorderly in the chloroplast, and thylakoid stacking was indistinct (Figures 5Ac,f), while abundant osmium particles also accumulated in the chloroplasts of the *pls4* mutant (Figures 5Ab,e).

Leaf variegation and necrotic lesions have been reported to result from ROS accumulation and usually result in cell apoptosis (Jiang et al., 2011). To further confirm leaf senescence by physiological and biochemical detection methods. ROS production in the leaves at the tillering and heading stages was measured via nitro blue tetrazolium (NBT) histochemical analysis. In the WT leaves, ROS were barely detected at both the tillering and heading stages; however, more stained areas appeared in *pls4* flag leaves than in WT flag leaves, especially at the heading stage (Figure 5B). These results are in accordance with the different degrees of the phenotypic differences during the two stages. The CAT and POD activity and concentrations of senescence-related substances, such as H_2_O_2_ and MDA, were subsequently measured. At both the tillering and heading stages, the concentrations of both H_2_O_2_ and MDA were higher in the *psl4* leaves than in the WT leaves (Figures 5C,D), but the activity of CAT and POD were lower in *pls4* mutant (Figures 5E,F). All of these indicators significantly differed at the heading stage. Therefore, these results suggested that the *pls4* mutants exhibits a more notable senescence phenotype than do the WT plants.

To understand the molecular basis for premature leaf senescence in *pls4* further, we assayed the expression levels of genes related to Chl degradation (i.e., *OsNOL, OsNYC1, OsNYC3, OsNYC4, OsPAO*, and *OsRCCR1*) *and* ROS scavenging (i.e., *OsCatB, OsPOD1, OsPOD2, OsAPX1*, and *OsAPX2*) in both WT and *pls4*. The expression of all genes related to Chl degradation was upregulated in *pls4* (Figure 5G). *OsCatB*, *OsPOD1*, *OsPOD2*, *OsAPX1*, and *OsAPX2* are genes associated with ROS scavenging. Unlike the expression levels of *OsAPX1* and *OsAPX2*, compared with those in the WT plants, the *OsCatB*, *OsPOD1*, and *OsPOD2* expression levels in the *pls4* mutants were downregulated. In particular, the *OsPOD1* transcriptional level in the *pls4* mutants was approximately 10 times less than that in the WT plants (Figure 5H). *OsAPX1* and *OsAPX2* are two ascorbate peroxidases genes, APX is a antioxidant enzyme of ROS scavenging in plant, O_2_^–^ and H_2_O_2_ were higher in the *pls4* leaves than the WT leaves (Figures 5B,C), which induced the *OsAPX* gene to be slightly upregulated in *pls4* mutant. Additionally, the expression of the common senescence marker *OsH36* (Lee et al., 2001), sharply increased in *pls4*, indicating that more severe senescence occurred in the mutant.

To determine which pathways were influenced in *pls4*, RNA sequence was performed by the tillering stage leaves, and the differentially expressed genes (DEGs) between *pls4* and WT were assayed. In total, we obtained 5867 DEGs, including 3091 upregulated and 2776 downregulated ones in *pls4* compared with WT, and the screening conditions were a false discovery rate (FDR) < 0.05 and a |log_2_FC| > 1. There were 485 DEGs, so pathway annotation was performed (Supplementary Table S5). Interestingly, DEGs between *pls4* and WT were mainly enriched in the lipid metabolic and senescence-associated pathway (Supplementary Table S6 and Supplementary Figure S7). These results indicated that the mutation of *OsPLS4* may affect wax biosynthesis and premature leaf senescence in the *pls4* mutant.

### *OsPLS4* Is Involved in Chilling Tolerance

**FIGURE 6:**
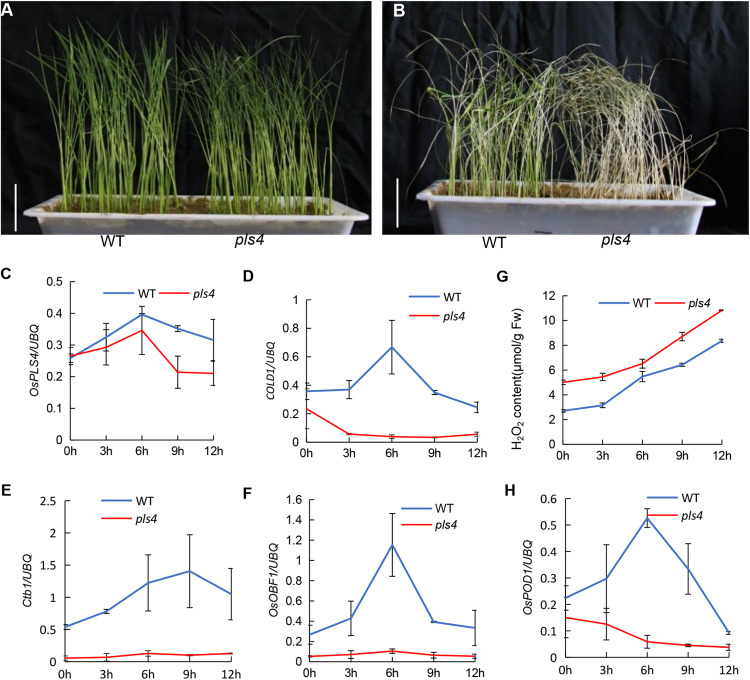
Chilling stress at the seedling stage and related gene expression analysis. **(A)** The WT and *pls4* mutant treated at 4°C for 72 h. Bar = 5 cm. **(B)** The WT and *pls4* mutant treated at 4°C for 72 h, followed by recovery at 30°C/25°C day/night for 7 days. Bar = 5 cm. **(C–F)** Relative expression of *OsPLS4* and genes associated with cold tolerance at different chilling treatment times for the WT and *pls4* mutant. The genes included *COLD1 (LOC_Os04g51180), Ctb1 (LOC_Os04g52830)* and *OsOBF (LOC_Os12g37410).* **(G)** H_2_O_2_ content in the WT and *pls4* mutant at different chilling treatment times. **(H)** Relative expression of *OsPOD1* (peroxidases) in the WT and *pls4* mutant at different chilling treatment times. The data presented are the means ± SDs from three biological replicates.

It is well known that plant cuticular wax provides an essential barrier for preventing abiotic and biotic damage, and stresses are important factors leading to senescence. In this study, we tested the impact of wax scarcity on the cold response. Three-leaf-stage seedlings of both *pls4* and WT were treated at 4°C for 72 h, and the leaves of the mutant became rolled and withered (Figure 6A). The treated seedlings were subsequently incubated at 30°C for 7 days for recovery. Eighty-two percent of the WT seedlings survived, while only a few *pls4* seedlings turned green, and most of them withered (Figure 6B). This result indicated that *pls4* is more sensitive than WT to cold stress. To explore the molecular mechanism by which *OsPLS4* regulates chilling tolerance further, the expression of several key cold tolerance-associated genes was measured every 3 h in treated seedlings. As such, the expression of *COLD1*, *Ctb1* and *OsOBF1* was measured. The expression of all three genes was obviously suppressed in *pls4*, as their expression was hardly detected. After low-temperature treatment, the transcript levels of *COLD1, Ctb1 and OsOBF1* peaked at 6 or 9 h after chilling but then decreased in the WT plants (Figures 6D–F). According to the chilling process, the *OsPLS4* expression level was similar to that of cold tolerance-associated genes and was higher in the WT than in *pls4* (Figure 6C). The results indicated that *OsPLS4* responded to low-temperature stress. Furthermore, the mutation of *OsPLS4* reduced fatty acid synthesis in the *pls4* plants and affect membrane fluidity in plant cells, which is also a key factor in low temperature sensitivity.

In addition, the more H_2_O_2_ accumulated in the *pls4* mutant than in the WT in all cold treatments, and as time progressed, the content of H_2_O_2_ also increased. Moreover, the expression of *OsPOD1*, which is essential to scavenging H_2_O_2_, increased after the first decrease, and in *pls4*, this gene expression level was always low (Figures 6G,H). The results again proved that the *pls4* plants were more sensitive to chilling stress than the WT plants.

## Discussion

To data, we propose that epicuticular wax crystals were significantly reduced on the mutant leaves by one base substitution in *OsPLS4*. However, the destruction of the cuticle synthesis pathway causes a senescence phenotype at the later growth stage. Leaf yellowing was the most obvious phenotype at the heading stage. This phenotype was confirmed by the disintegration of chloroplasts, reduction in Chl content and photosynthetic rate, the higher accumulation of ROS and MDA and up-regulation of SAGs. In addition, cuticular wax defect caused that the *pls4* plants were more sensitive to chilling stress than the WT plants.

Premature leaf senescence severely affects the growth and yield of rice, and leaf cells undergo different metabolic changes during senescence (Hörtensteiner, 2006; Schippers et al., 2015). In the last decade, a series of leaf SAGs have been identified and characterized in rice by using different leaf senescence mutants (Jiao et al., 2012; Liang et al., 2014; Sakuraba et al., 2015; Hong et al., 2018). However, the molecular mechanism controlling leaf senescence remains poorly understood. In this study, we have identified *OsPLS4* gene from the *pls4* mutant via map-based cloning and a genomic complementation assay. The *pls4* mutant differed from previously reported early senescence mutants (Liang et al., 2014; Yang et al., 2016; Leng et al., 2017a). The change of leaves color and reduction of Chl content mainly occurred during the heading stage (Figures 1B,D). TEM revealed that the number and size of chloroplasts were dramatically reduced in yellow leaves of the *pls4* during the heading stage, and the granum thylakoids (GTKs) were arranged disorderly in the chloroplast of the *pls4* mutant (Figures 5Aa–f). These results indicated that the ultrastructure of chloroplasts was disrupted in the *pls4* mutant. In previous studies, SAGs were involved mainly in chloroplast development and degradation, protein synthesis, degradation and transport pathways, the hormone pathway and the programmed cell death pathway among others (Leng et al., 2017b; Hong et al., 2018). However, we isolated and cloned a rice *OsPLS4* gene (Figure 2), encoding a putative 3-oxoacyl-ACP reductase, which is a key enzyme involved in fatty acid biosynthesis found in many plant chloroplasts (Joyard et al., 1998; Heredia-Martínez et al., 2018). Moreover, fatty acids are synthesized mainly in thylakoid membranes and are major components of membrane lipids (Joyard et al., 1998; Li et al., 2016). Hence, affected fatty acid synthesis pathways can result in membrane lipids degradation, altering membrane composition, and disrupting membrane fluidity and permeability in chloroplast (Dietz et al., 2016; Li et al., 2016; Zhang et al., 2016). Interesting, our results showed that the OsPLS4 protein was located in the chloroplast, which is consistent with its putative biochemical function (Figure 3C). TEM showed abnormal chloroplast development, with indistinct thylakoid stacking and abundant accumulation of osmium particles in the *pls4* mutant (Figure 5A). The Chl content and photosynthetic rate dramatically decreased in *pls4* (Figures 1D,E), which both are indicator of premature leaf senescence. Taken together, these results demonstrate that the mutation of *OsPLS4* indirectly affected chloroplast normal development and further caused leaf senescence.

Some important changes are observed in premature leaf senescence. For instance, leaf color, degradation of chloroplasts, reduction in Chl content and photosynthesis, accumulation of ROS, reduction of yield-related attributes (Rani et al., 2002; Khanna-chopra, 2012; Huang et al., 2016; Leng et al., 2017b). In addition, cuticle is strongly linked with the release of ROS, Under conditions where the cuticular barrier is broken, ROS are induced (L’Haridon et al., 2011). In our study, ROS accumulation was obvious increased in the *pls4* mutant with reduced surface cuticle. NBT staining exhibited that H_2_O_2_ production in leaf segments occurred at higher levels in the *pls4* mutant than in the WT (Figure 5B), and the concentrations of both H_2_O_2_ and MDA increased significantly in the leaves of *pls4*, whereas the scavenging enzymes CAT and POD activity decreased (Figures 5C–F). These results suggested that the *pls4* plants responded actively to the H_2_O_2_ and MDA accumulation but the scavenger CAT and POD activity did not increase and execution of senescence phenotype in the *pls4* mutant leaves. However, high ROS accumulation cannot been eliminated effectively in plant cell, which damages to thylakoid membranes and other cellular components. Compared with the tillering stage, all these senescence indicators have reached the extremely significant difference at the heading stage (^∗∗^*P* < 0.01) (Figures 5B–D), whose high ROS accumulation can explain why the *pls4* plants exhibited yellow leaf and Chl content rapidly decreased after heading, but not at the previous stages. According to the expression pattern of *OsPLS4*, the *OsPLS4* expression level was relatively low in the leaves before the tillering stage, and the *OsPLS4* expression level was the highest in the leaves at the tillering stage (Figure 3A), which indicated the *OsPLS4* gene may play an important role in the tillering stage. Moreover, RNA-seq analysis showed that DEGs were mainly distributed in lipid metabolism and senescence related pathway, which may cause lipid metabolic disorders, altering the cuticle synthesis and leaf senescence of the *pls4* mutant. Taken together, these factors may be a reason why the plant height and size of the *pls4* mutant reduced compared with WT at the tillering stage and yellow leaf after the flowering. 3-oxoacyl-reductase activity and the molecular regulation mechanism of the *OsPLS4* will be further studied to better explain why the mutants exhibit leaf senescence at the heading stage.

The plant cuticular wax biosynthesis process is very complex and begins with fatty acid synthesis in the plastid, followed by assembly in the ER (Kunst and Samuels, 2003; Karki et al., 2019). Several genes have been identified in rice by the characterization of mutants whose leaves are sparsely covered with wax crystals, which are involved in VLCFA elongation of the fatty acid synthesis pathway in the ER, and the subcellular localization of these proteins was mostly in the ER (Yu et al., 2008; Mao et al., 2012; Gan et al., 2016; Wang et al., 2017). In this study, SEM and GC-MS analysis showed leaves surface wax content significant decrease in the *pls4* mutant (Figures 4A–D,G and Table 1). However, the subcellular localization of the PLS4 protein was in the chloroplast (Figure 3C), and we speculated that *OsPLS4* was involved in the first step of fatty acid synthesis and indirectly affects cuticular wax synthesis. To verify the *OsPLS4* function, we determined that the expression levels of wax synthesis-related genes were downregulated in *pls4* (Figure 4G), with the exception of *OsKCS1* and *OsFATB1*, which may be due to the *OsPLS4* mutation leading to a reduction in intermediate products during the initial stage of fatty acid biosynthesis. In addition, analysis of the water-loss rate and Chl leakage ratio indicated that the leaf surface of the *pls4* mutant had less wax than did the leaf surface of the WT (Figures 4E,F). Taken together, these results demonstrate that *OsPLS4* is involved in fatty acid synthesis and regulates cuticular wax synthesis in rice.

Plant cuticular wax is the initial protective barrier against the external environment, with a key role in protecting plants from abiotic and biotic damage (Kunst and Samuels, 2003). In plants, the fluidity and stability of membranes is important to sustain the functional activity of membrane proteins and the membranes themselves, which are closely related to temperature; cold-tolerant plants contain a greater abundance of unsaturated fatty acids (Yu et al., 2009; Tovuu et al., 2016; Wang et al., 2019). Moreover, chilling stress mediates a series of physiological and metabolite changes, such as alterations in chlorophyll fluorescence, H_2_O_2_, ROS, lipid peroxides, and other metabolites (Wang et al., 2010; Liu et al., 2018). In the present study, we found that the temperature remained low for several days, and leaf senescence was more obvious in the *pls4* mutant than in the WT in the field. Moreover, we subjected plants at the seedling stage to low temperature. The results indicated that the *pls4* plants were more sensitive to chilling stress than were the WT plants (Figures 6A,B). Furthermore, the H_2_O_2_ contents in the *pls4* mutant were higher than those in the WT under chilling stress (Figure 6G), which further confirmed this conclusion. The variation trend of *OsPLS4* was similar to that of the chilling tolerance-associated genes (Figures 6C–F), confirming that *OsPLS4* plays an important role in the clod tolerance of rice. Thus, we demonstrate that *OsPLS4* responds to chilling stress by regulating fatty acid synthesis. However, the molecular mechanism controlling chilling stress needs to be further examined with the use of *OsPLS4* over-expression plants.

Previous reports showed that decreased photosynthesis leads to a lack of biomass production in plant leaves, which can cause an inadequate supply of nutrients for the rice grain at the filling stage; premature senescent leaves have a substantial influence on grain filling and further affect rice yield. In this study, leaf senescence and low photosynthetic rate of the *pls4* mutant affects normal plant growth and the grain-filling rate, eventually resulting in decreased rice grain production and relatively low grain quality (Supplementary Tables S3, S4), which is consistent with the previous results of reports (Yang et al., 2016; Hong et al., 2018). In addition, our results showed that pollen fertility and the seed setting rate obviously decreased in *pls4* mutant (Supplementary Figure S2 and Supplementary Table S3). These altered agronomic traits ultimately affected the grain yield. Overall, these results indicated that cuticular wax is not only essential for protecting rice plants from external damage but also necessary for plant growth and development.

## Conclusion

**FIGURE 7:**
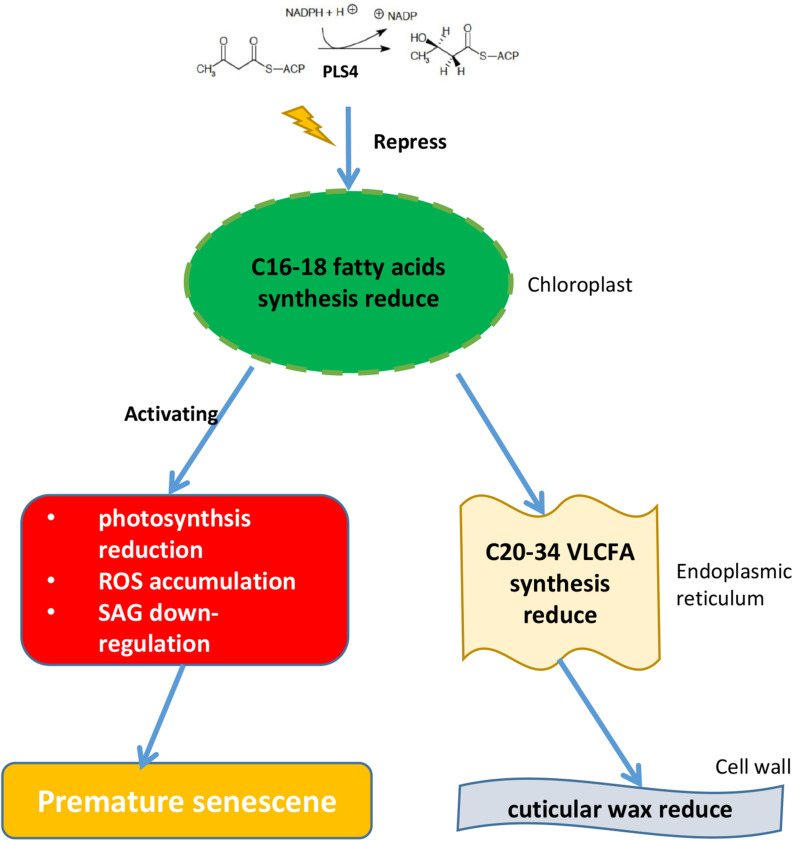
Proposed model for the function of *OsPLS4* in cuticular wax formation and leaf senescence. *OsPLS4* gene mutation would lead to blocked fatty acid synthesis in chloroplast, which caused the VLCFA synthesis and cuticular wax decrease in *pls4* mutant. However, abnormal chloroplast development affected the photosynthetic rate reduction, ROS accumulation and the expression level of the SAGs, ultimately leading to premature leaf senescence in the *pls4* mutant.

A simple mechanism was proposed to explain *OsPLS4*-mediated cuticular wax biosynthesis and leaf senescence in rice (Figure 7). We confirmed that *OsPLS4* was involved in fatty acid synthesis in the chloroplast, and amino acid change of OsPLS4 inhibits fatty acid synthesis to caused chloroplast defective development and weaken photosynthesis, ultimately leading to premature leaf senescence in the *pls4* mutant. This senescence mechanism differs from previously reported mechanisms based on genes directly involved in chloroplast synthesis and degradation. Furthermore, decreases in cuticular wax caused the *pls4* mutant plants to be more sensitive to chilling stress than the WT plants. Therefore, our study provides insight into new molecular mechanisms involved in premature leaf senescence and theoretical basis for plant stress resistance.

## Data Availability Statement

The original contributions presented in the study are included in the article/Supplementary Material, further inquiries can be directed to the corresponding authors.

## Author Contributions

HH, WT, and JX conceived and designed the research. TL, YY, LO, and LP carried out the physiological characterization of the mutants and map-based cloning of *OsPLS4*. DZ, ZQ, JF, and XL performed the plant physiological and chilling treatment experiment. DZ, JX, and ZJ carried out the RNA-seq, confocal microscopy, and bioinformatics data analyses. DZ and JX wrote the manuscript. CZ, XP, and JB supervised the experiments. All authors reviewed the manuscript.

## Conflict of Interest

The authors declare that the research was conducted in the absence of any commercial or financial relationships that could be construed as a potential conflict of interest.

## Footnotes

1 http://www.gramene.org
