# Supplementary material for: OsPLS4 Is Involved in Cuticular Wax Biosynthesis and Affects Leaf Senescence in Rice
Source: Front Plant Sci. 2020 Jun 11;11:782. doi: 10.3389/fpls.2020.00782 (PMC7300252; doi:10.3389/fpls.2020.00782)
Supplement: Supplementary file 1 [file Data_Sheet_1.pdf]

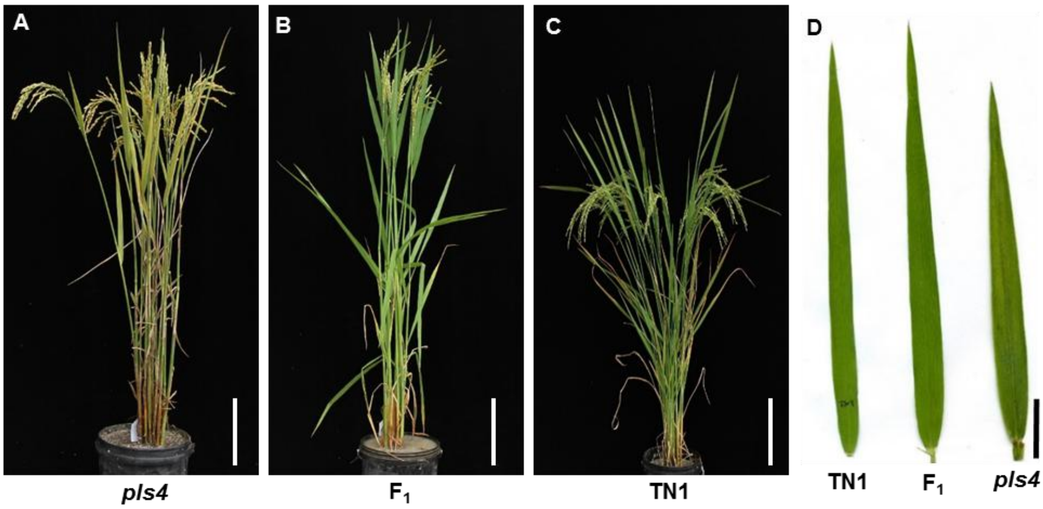

Figure S1. The *pls4* mutant, TN1 and F_1 phenotype.
(A-C) The plant phenotype. **(D)** Plants correspond to leaf phenotype. Bars: (A-C) 10 cm; (D) 1 cm.

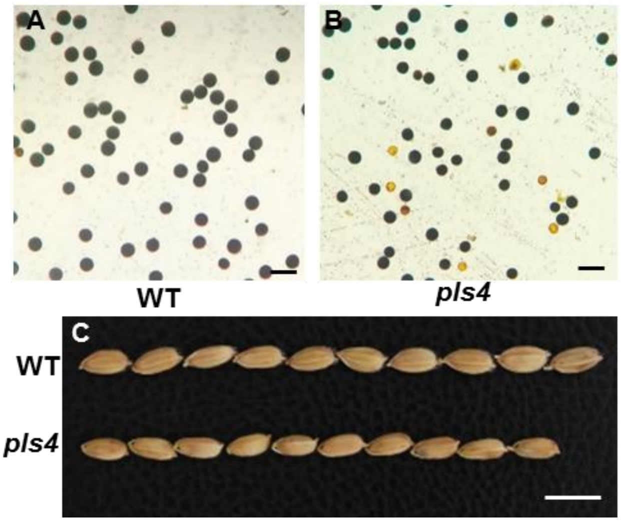

Figure S2. Pollen fertility analysis and grains size of WT and *pls4*.
(A-B) I_2-KI staining of WT and *pls4* mature pollen grains. **(C)** The grains size of WT and *pls4*. Bars: (A, B) 1 mm; (C) 1 cm.

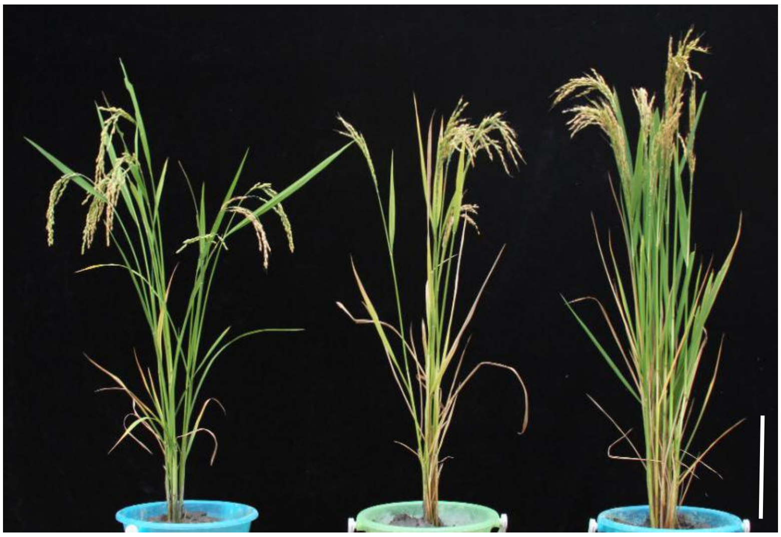

Figure S3. WT, *pls4* mutant and complementation plants.
OsPLS4-complementation plants were developed by transforming the whole genomic fragment of *OsPLS4* into the pls4 mutant. Bar=10 cm.

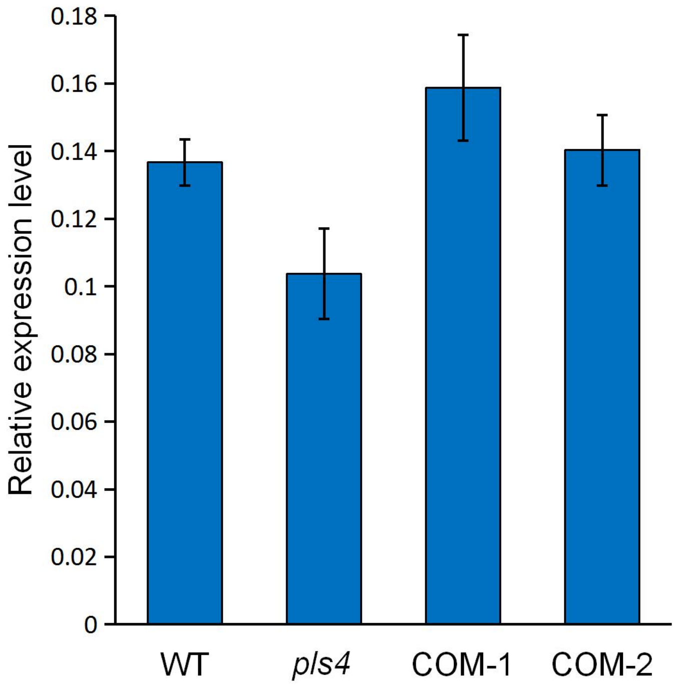

Figure S4. qPCR analysis of *PLS4* expression in the tillering leaves of WT, *pls4* and two COM independent lines. The data presented are the means±SDs of three biological replicates.

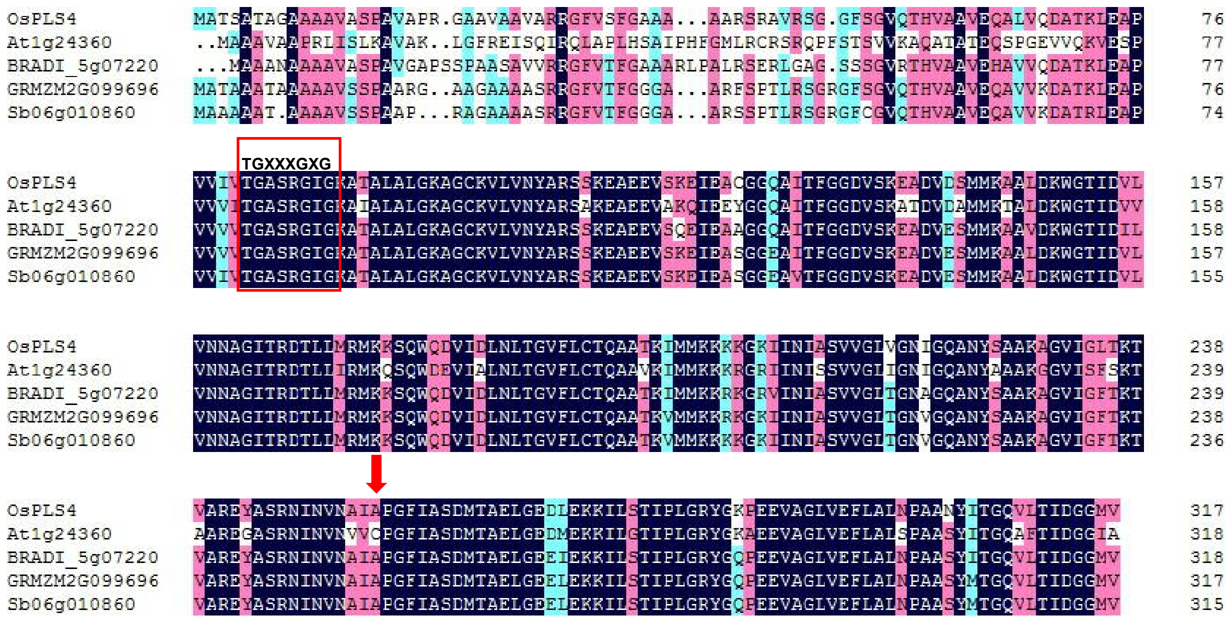

Figure S5. OsPLS4 protein sequence alignment of homologues.

Protein sequence alignment of *OsPLS4* and its homologues in sorghum, maize, Brachypodium and *Arabidopsis* according to Clustal W. Conserved amino acids are highlighted: white letters on a black background indicate amino acids that are conserved across all these samples. The arrowhead shows the mutant amino acid in the protein of the *pls4* mutant, Red box contains TGXXXGXG is classical-SDR NAD-binding motif.

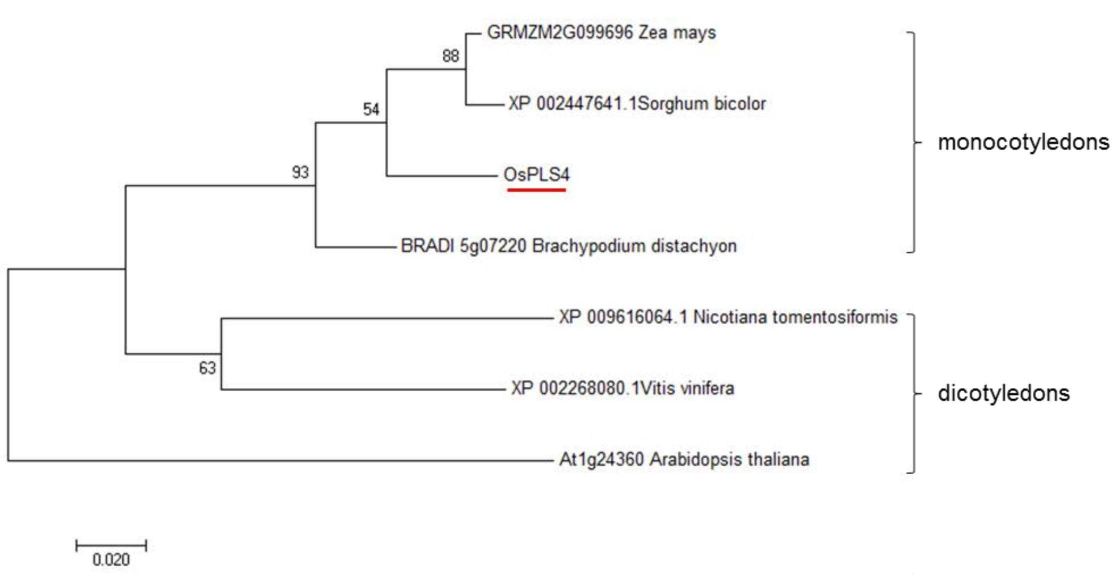

Figure S6. Phylogenetic tree analysis of OsPLS4 using the maximum likelihood method from MEGA7 software.

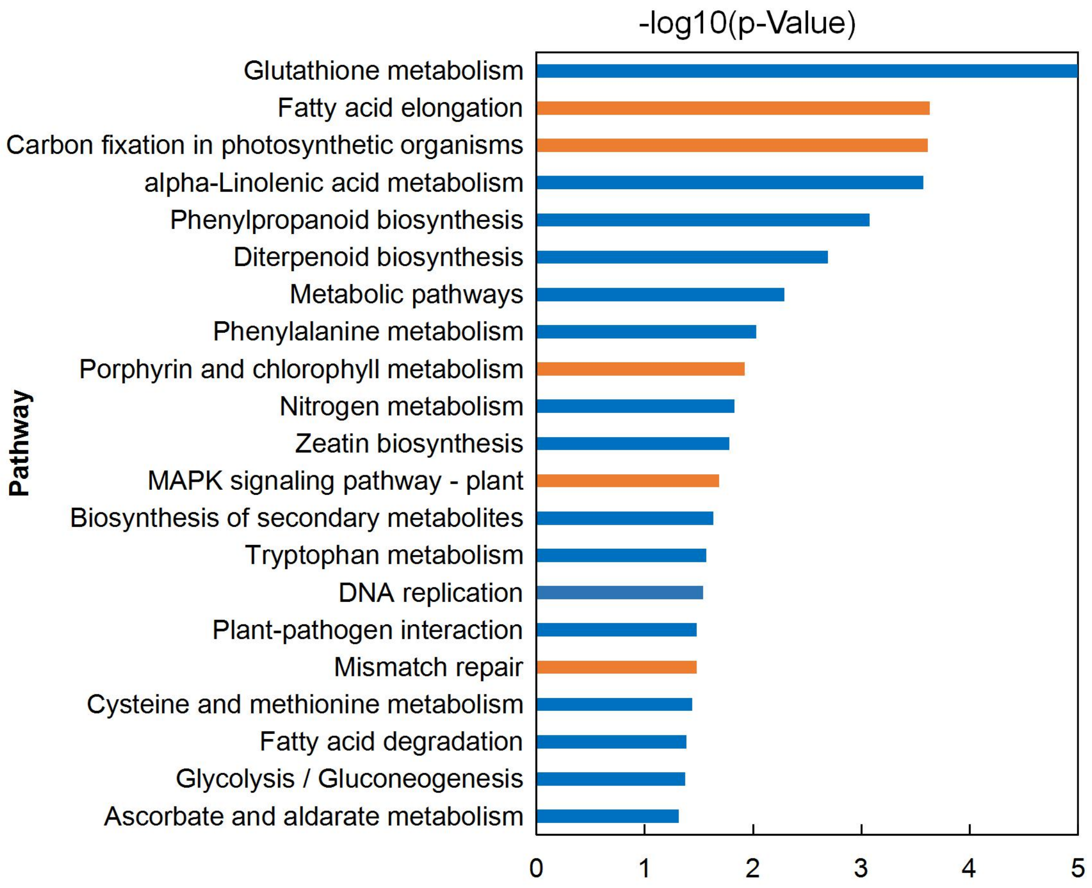

Figure S7. KEGG enrichment analysis shared between the WT and *pls4*.
This figure is illustrates the top 20 enriched of pathways and P-value sequencing from small to large. Orange represents some of the biological pathways associated with this study.

Table S1. Genetic analysis of F_2 populations derived from crosses between *pls4* and other conventional varieties

Combination	F_1		F_2		χ^2	P-value
	Normal	Leaf senescence	Normal	Leaf senescence		
pls4/Nip	10	0	363	112	0.534702773	0.47445614
Nip/pls4	6	0	218	65	0.664425342	0.429902822
pls4/TN1	9	0	286	87	0.588627854	0.454851356
TN1/*pls4*	8	0	267	76	1.620355896	0.224066686
pls4/9311	8	0	245	69	1.691198191	0.215676424
9311/*pls4*	7	0	225	70	0.264452179	0.61410715

Table S2. Primers.

Reaction	Primer name	Forward primer (5'-3')	Reverse prime (5'-3')
Gene mapping	M1	TATGCGAAGGATGTGCGAC	ACGAATACATGTGCCTGCC
	M5	CGGAGCTGGTCTAGCCATC	GTCTCCGTCTTCCTCACTCG
	M6	AATGGGACCAGAAAGCACAC	AAAAAGAGCATGGGGGCTAC
	M3	AACCGGCCATGCCAGAGAGG	CCAAATCCTATCCGCCACACACC
	M2	CTGGATCTGTGAGGTGTCTCTAGG	AAAGAGGAGTTCGCACAAGTGG
	M4	GTAGCCTTGCACTCGACCGTACC	ACCAACTCTGGCAATGCATCC
	C1	CATTGCCAACCCGTAAAGCTACC	GGTGAGCTGAAGATGTTCTTTCATGG
	C2	CCCTGCACCTGGATTCTCTCTCC	ATTGACGCACAGACAAGAACAAGACG
	C3	TTATTAGAGCGCCATGTGGC	CATGCTGTGGTTTGTCAAGG
	C4	AGCAACTGCACAGGAATAAT	ATGTGCCACCATAAGTTGAT
	C7	CGTTCAAGGAGCTTGTGTTGATCC	GGACCGATTTAAGTGAACGTTGATGG
	C8	TTCCACAACCCTTGTATGTGTGC	GTGCATGAGAAGAGGATAGATGTGG
	C9	CGACACAAGCACTAGGCATC	TATGTCGAGGACGATGGACA
	C10	TGTCCACATCGCCATGTACT	TGAAGGACGCTACGGAACTC
Subcellular localization	PLS4-GFP	GACAGCCCAGATCAACTAGTATGGCGAC CTCCGCGACCGCAG	GCCCTTGCTCACCATGGATCCCATCACCA TCCCTCCATCG
Construction of genetic complementation vector	PLS4-COM	GAGCTCGGTACCCGGGGATCCTGTATGC CCAATAAAAACACTTTGG	ACGACGGCCAGTGCCAAGCTTTACTTCC TTCATGCATTCATCTCC
Over-expression	PLS4-OE	GAATTCATGGCGACCTCCGCGACCGCAG GGGCAGCAG	GAATTCCTACATCACCATCCCTCCATCGA TGGTAAG
GUS reporting vector	PLS4-GUS	GCTATGACCATGATTACGAATTCACTTGT TAATGATATTTCTTGGATT	CTAGAAATTTACCCTCAGATCTACCATGA GTGCGCGTAGAGAAGACGC
Gene expression analysis	*OsUBQ(Ubiqutin)*	ACCCTGGCTGACTACAACATC	AGTTGACAGCCCTAGGGTG
	OsActin	TGGCATCTCTCAGCACATTCC	TGCACAATGGATGGGTCAGA
	OsPLS4-rt	CTCTGTACACAAGCTGCTACAA	ATTACCAACAAGACCAACAACT
	OsWR1	AGAAGTCCCACATTGGCGTGT	GCTCAGCAACTCCTCGATCATT
	OsCER7	ACCACCATCTGTGGTTGAGGACAA	TTGGCGATATAGCTTCCTGCGTCT
	OsCER10	CGAGTGGTAACGGTGGCTAT	CTGTGTGGCAATGTTGAACC
	OsFDH2	TGTGAACAATGCTCGTGGAG	CCTGTCCAGCATGTCCTTTA
	OsKCS2	GTACAGGTTTGGGAACACGTCG	GCTCAGTCGGTTGAAATCCTGG
	OsMAH1	ACCACGTCAAGTCGAAGCAGAAGA	AGTTCAACGGTGTGACTGTGAGGA
	OsKCR1	ACCCGCTCTACAGCGTCTAC	TACAGGGGTACCTGGCATTG
	OsPAS2	TGCATCCATCCTTTCTCTCGCCAT	CCTTTGCCAATGCCTTCTTCCGTT
	OsWSL3	CAATTCTCGAGATGCCTCTATGT	TCTTGATGGACGCCATCTTC
	OsCatB	GCTTGCTTTCTGCCCAGCGATAAT	AAATAGTTTGGGCCAAGACGGTGC
	OsPOD1	ACGTCGGGGTCGCCAACAAC	CGAACTCGTCCACCGACGCC
	OsPOD2	AGGCTCAACTGCTCCAGGGTCA	TGGCAATAAACCGGACAAGCCCT

OsAPX1	AGGTGCCACAAGGAAAGATCTGGT	TCAGCAGGGCTTTGTCACTAGGAA
OsAPX2	TGGGAAGATGCCACAAGGAGAGAT	TCCGCAGCATATTTCTCCACCAGT
OsRCCR1	GGATCGACGATTGATTTCATG	GTCGAGGCGTTCAGAAAGAT
OsH36	CCTGGTGATCTGAAGGTTGT	CATGGCAACCAGTGTAAAGC
OsNOL	CCACGAAAGGTATAGGATATG	TCAAGTCAGTCACCGCAGAT
OsNYC1	TTTTGAGCGGTTCTTCTCAGA	CCTTTCACAACTCGCATCCT
OsNYC3	TCTATCTAGGTGCCAAAGGC	ATTCTGGCACCTGCTGTTTC
OsNYC4	CGTCTATGACCAACTCATGG	TGCGTCAGCTCTGTATTGCT
OsPAO	AAGCCTCCGATGTTACCGAA	CGAGGGTTTCCAGAATTTGA
OsSGR	GAAGCTGCCGCGGACGTACA	GTTGCCGTCGCCGTGGAC
OsLhcb1	CCATGTTCTCCATGTTCGGCTTCT	TAGGCCCAGGCGTTGTTGTTGA
OsLhcb4	TACCTGCAGTTCGAGCTGGAC	AGGCCGAACACCTCGGTGTA
COLD1	TCTCCTGCAACCTCTTCCAG	TGGTAGTAGGGGAGGACGAA
OsCtb1	GGTGTGTCAAGTCAGAGGGA	CAACAGCAACTAGGGGAGGA
Osmyb4	TTCCAGATCGACGACAGCTT	GGACTCCATGAACACTCCGA
OsOBF1	TGGAGCAGGAGAACACGG	CGCTGAACTCCTCGACGA

Table S3. Agronomic traits in wild-type and *pls4*.

Traits	Plant height(cm)	Tillering numbers	Panicle length(cm)	Grains per panicle	Seed-setting rate(%)	1000-grain weight
WT	91.40±1.74	9.48±1.16	18.14±0.71	118.67±27.78	96.10±0.96	26.57±0.30
pls4	89.56±2.00	9.40±1.02	16.26±0.76	107.37±11.78	68.18±7.33**	18.89±0.65**

Table S4. Rice quality related traits between wild-type and *pls4*.

Traits	WT	*pls4*
Degree of chalkiness(%)	3.85±0.14	4.56±0.23
Gel consistency（mm）	83.23±2.72	68.23±3.11**
Amylose content(%)	16.25±0.75	15.37±0.21
Gelatinization temperature	6	6

Table S5. WT-vs-*pls4* Pathway Enrichment.

Pathway	DEGs genes with pathway annotation (485)	All genes with pathway annotation (3233)	Pvalue	Qvalue	Pathway ID
Glutathione metabolism	24 (4.95%)	62 (1.92%)	0.000004	0.000416	ko00480
Fatty acid elongation	10 (2.06%)	20 (0.62%)	0.000236	0.007865	ko00062
Carbon fixation in photosynthetic organisms	21 (4.33%)	64 (1.98%)	0.000244	0.007865	ko00710
alpha-Linolenic acid metabolism	14 (2.89%)	35 (1.08%)	0.000269	0.007865	ko00592
Phenylpropanoid biosynthesis	31 (6.39%)	118 (3.65%)	0.000842	0.019695	ko00940
Diterpenoid biosynthesis	10 (2.06%)	25 (0.77%)	0.002052	0.040019	ko00904
Metabolic pathways	13 (2.68%)	41 (1.27%)	0.005178	0.086554	ko01100
Phenylalanine metabolism	10 (2.06%)	30 (0.93%)	0.009344	0.136658	ko00360
Porphyrin and chlorophyll metabolism	10 (2.06%)	31 (0.96%)	0.011995	0.155934	ko00860
Nitrogen metabolism	8 (1.65%)	23 (0.71%)	0.014879	0.174081	ko00910
Zeatin biosynthesis	6 (1.24%)	15 (0.46%)	0.016582	0.176367	ko00908
MAPK signaling pathway - plant	20 (4.12%)	84 (2.6%)	0.020668	0.20151	ko04016
Biosynthesis of secondary metabolites	6 (1.24%)	16 (0.49%)	0.02325	0.20925	ko01110
Tryptophan metabolism	9 (1.86%)	30 (0.93%)	0.027181	0.22695	ko00380
DNA replication	11 (2.27%)	40 (1.24%)	0.029096	0.22695	ko03030
Plant-pathogen interaction	24 (4.95%)	110 (3.4%)	0.03302	0.229828	ko04626
Mismatch repair	9 (1.86%)	31 (0.96%)	0.033394	0.229828	ko03430
Cysteine and methionine metabolism	18 (3.71%)	78 (2.41%)	0.036577	0.237751	ko00270
Fatty acid degradation	10 (2.06%)	37 (1.14%)	0.040978	0.248564	ko00071
Glycolysis / Gluconeogenesis	23 (4.74%)	107 (3.31%)	0.04249	0.248564	ko00010
Ascorbate and aldarate metabolism	10 (2.06%)	38 (1.18%)	0.048459	0.269987	ko00053
Flavonoid biosynthesis	6 (1.24%)	19 (0.59%)	0.053186	0.273055	ko00941

Table S6. Senescence-related genes identified by RNA-seq analysis are agreement with the expression patterns of RT-qPCR.

Locus	Annotation	Fold change
Up-regulation		Log2 (FC)
LOC_Os01g12710	Chlorophyll b reductase, Leaf senescence	0.4375
LOC_Os06g24730	Chlorophyll degradation during senescence	0.7291
LOC_Os03g05310	Pheophorbide a oxygenase, Leaf senescence	1.0768
LOC_Os10g25030	Red chlorophyll catabolite reductase, Leaf senescence	2.1437
LOC_Os06g51150	Catalase isozyme B	0.5323
LOC_Os03g17690	Similar to L-ascorbate peroxidase	0.8026
LOC_Os07g49400	L-ascorbate peroxidase	0.7896
LOC_Os05g39770	Similar to Alanine:glyoxylate aminotransferase-like protein	2.5450
